# Approach-avoidance sequencing shapes embodied simulation of interpersonal closeness-distance: evidence from second-person episodes in Spanish and Chinese

**DOI:** 10.3389/fpsyg.2026.1925565

**Published:** 2026-08-21

**Authors:** Yuxiao Sun, Huili Wang, Iván Padrón, Hipólito Marrero

**Affiliations:** 1 Departamento de Psicología Cognitiva, Social y Organizacional, Universidad de La Laguna, San Cristóbal de La Laguna, Spain; 2 Instituto Universitario de Neurociencia (IUNE), Universidad de La Laguna, San Cristóbal de La Laguna, Spain; 3 School of Foreign Languages, Hangzhou City University, Hangzhou, China; 4 Hangzhou Collaborative Innovation Institute of Language Services, Hangzhou, China; 5 Psicología Evolutiva y de la Educación, Universidad de La Laguna, San Cristóbal de La Laguna, Spain; 6 Instituto de Investigación Sanitaria de Canarias (IISC), Gobierno de Canarias, La Laguna, Spain

**Keywords:** approach-avoidance motivation, cross-linguistic comparison, embodied simulation, interpersonal distance, second-person perspective

## Abstract

We investigated how approach and avoidance motivational sequences shape evaluations of interpersonal closeness-distance during second-person verbally simulated interaction episodes, and whether these effects are modulated by linguistic formulation and language context. Two parallel behavioral experiments were conducted in Spanish (*N* = 20) and Mandarin Chinese (*N* = 20; 39 participants retained for analysis after one exclusion). Participants read fictional interaction episodes in which they adopted the role of the protagonist and interacted with a character through three consecutive approach- or avoidance-oriented behaviors. Episodes followed either an approach-avoidance-approach (Ap_Av_Ap) or avoidance-approach-avoidance (Av_Ap_Av) sequence and were expressed using either attitudinal formulations (e.g., “Juan asked you for he being a part of your class group and you *accepted-rejected* him”) or equivalent behavioral formulations (e.g., you *said yes*, *said no* to him). Following each episode, participants evaluated perceived interpersonal closeness-distance toward the character. Linear mixed-effects analyses revealed a robust effect of motivational sequence: Ap_Av_Ap episodes elicited greater perceived interpersonal closeness than Av_Ap_Av episodes across both language groups and linguistic formulations. Attitudinal formulations facilitated sentence processing, yielding shorter reading times than behavioral formulations, although linguistic formulation did not influence final interpersonal evaluations. Sequence effects emerged during earlier sentence processing but disappeared at the final sentence, consistent with dynamic updating of interpersonal representations across the episode. Cross-linguistic differences were also observed: Chinese participants showed overall faster reading times and rated episodes as involving greater interpersonal closeness than Spanish participants. These findings support an embodied and language-mediated account of interpersonal cognition in which readers dynamically integrate approach and avoidance information to construct evaluations of interpersonal closeness-distance during second-person interaction simulation. The results further suggest that motivational sequence effects generalize across languages while remaining sensitive to cultural and linguistic modulation.

## 1 Introduction

Social interactions are fundamental to human life and are shaped by behavioral tendencies that reflect basic motivational orientations: approach and avoidance. These motivational systems, widely studied in social psychology and cognitive neuroscience, guide responses to social stimuli according to their perceived affective value and are expressed not only in overt behavior but also in cognition and emotion. Approach-oriented tendencies typically promote affiliation, acceptance, cooperation, and relational closeness, whereas avoidance-oriented tendencies are associated with rejection, disengagement, conflict, or interpersonal distancing (Corr, 2013; Elliot, 2006; Elliot et al., 2025; Gable, 2006; Gable and Gosnell, 2013; Marrero et al., 2015, 2023a, 2023b). Together, these orientations provide a motivational framework through which individuals regulate social behavior and navigate interpersonal relationships.

Language plays a central role in representing and coordinating interpersonal cognition (Corballis, 2017; Fusaroli et al., 2014; Seyfarth and Cheney, 2014; Tylén et al., 2010). According to embodied simulation theories, understanding narrated actions, emotions, and social interactions involves the reactivation of sensorimotor and affective experiences associated with those events (Barsalou, 1999, 2008; Bechtold et al., 2023; Gallese, 2006, 2009; Morey et al., 2022; Zwaan, 2004). During language comprehension, readers construct mental representations by simulating the actions, intentions, and emotional consequences described in the text. We adopt this embodied simulation framework as the guiding theoretical lens of the present study: if interpersonal meaning is grounded in simulated experience, then both the perspective from which an episode is read and the order in which its actions unfold should shape how interpersonal closeness and distance are represented.

This process may be particularly relevant in second-person narratives, in which readers adopt the protagonist’s perspective and mentally place themselves within the interaction (Redcay and Schilbach, 2019). Whereas third-person constructions typically promote a more observer-based representation of another individual’s behavior, second-person narratives may foster a more self-involving and egocentric simulation by mapping actions more directly onto the reader’s own bodily and affective systems (Barsalou, 1999, 2008; Glenberg and Kaschak, 2002; Zwaan, 2004). Consistent with this view, previous work has shown that approach and avoidance behaviors embedded in narrative contexts evoke distinct cognitive patterns during comprehension, supporting the assumption that interpersonal interactions are mentally represented through embodied simulation mechanisms (Marrero et al., 2015, 2017, 2023a, 2023b).

From an embodied perspective, interpersonal closeness and distance may be represented using spatial schemas originally developed for physical navigation. The common-origin hypothesis proposes that social and spatial distance partially rely on overlapping cognitive and neural mechanisms, such that judgments of social closeness recruit representational systems also involved in spatial proximity (Anderson, 2010; Yamakawa et al., 2009). Everyday linguistic expressions such as *close friend* or *distant relative* further suggest that interpersonal relations are conceptualized through spatial metaphors. Within this framework, approach-oriented interactions may be mentally represented as movements toward interpersonal proximity, whereas avoidance-oriented interactions may be represented as movements toward interpersonal distance (Marrero et al., 2015, 2023a, 2023b).

Importantly, language may influence how these motivational orientations are cognitively represented. Interpersonal actions can be formulated using attitudinal verbs that explicitly encode intentional stance (e.g., *accept*, *reject*, *support*) or using more neutral behavioral expressions that describe the action without directly specifying attitude (e.g., *say yes*, *say no*). Although both formulations may describe functionally equivalent interpersonal behaviors, attitudinal verbs provide more direct access to motivational and affective meaning, whereas behavioral formulations may require readers to infer intentional stance from contextual information. Consequently, linguistic formulation may influence the efficiency with which motivational information is processed and integrated during interpersonal simulation.

In natural social interactions, however, interpersonal meaning rarely depends on isolated behaviors. Rather, relationships unfold across temporally ordered sequences of actions. The motivational arrangement of approach and avoidance behaviors within an interaction episode may therefore shape how interpersonal closeness or distance is ultimately evaluated. For example, an episode that begins and ends with approach behaviors (approach-avoidance-approach; Ap_Av_Ap) may generate a different relational perception than one dominated by avoidance (avoidance-approach-avoidance; Av_Ap_Av). How such temporally distributed information is combined is addressed by two closely related accounts of narrative comprehension. Situation-model theory holds that readers do not retain text verbatim but build a mental model of the described situation, which they continuously update along dimensions such as space, time, causality, and the protagonist’s goals as new information arrives (Zwaan and Radvansky, 1998; Gámez and Marrero, 2001). Event-segmentation theory specifies the dynamics of this updating: comprehenders parse the ongoing stream of actions into discrete events and revise their current event model at event boundaries, where incoming information no longer fits the active representation (Kurby and Zacks, 2012; Zacks et al., 2007). Such boundaries have been established through converging evidence, including agreement in explicit segmentation judgments, increased reading times and processing costs at boundaries, enhanced memory for boundary information, and characteristic neural responses at points of representational change (Bailey et al., 2017; Zacks et al., 2007). Although these accounts emphasize the temporal dynamics of comprehension whereas embodied simulation emphasizes its grounded, experiential substrate, they share a core assumption: meaning is constructed incrementally rather than read off isolated units, with readers building and revising a coherent representation as events unfold. Integrating the two, we propose that interpersonal closeness-distance is computed by simulating each approach or avoidance interaction and updating a grounded relational representation across the episode, so that the final evaluation reflects the cumulative, sequentially integrated affective meaning of the interaction rather than any single behavior.

Despite extensive work on approach-avoidance motivation, on embodied simulation in language, and on event-based narrative updating, these literatures have largely developed in parallel. It remains unknown whether the temporal sequencing of approach and avoidance interactions is dynamically integrated into interpersonal closeness-distance judgments, and whether such integration is modulated by linguistic formulation or generalizes across typologically distinct languages. The present study addresses this gap by investigating how motivational sequencing in second-person verbally simulated interaction episodes influences evaluations of interpersonal affective closeness-distance, and whether this process is modulated by linguistic formulation and language context. Participants read short fictional episodes in which they adopted the role of the protagonist and interacted with a character through three consecutive approach or avoidance behaviors. Episodes followed one of two motivational sequences—Ap_Av_Ap or Av_Ap_Av—and were expressed either through attitudinal verbs or equivalent behavioral formulations. After each episode, participants evaluated perceived interpersonal closeness-distance toward the fictional character.

In addition, we examined whether these processes generalize across linguistic and cultural contexts by conducting parallel behavioral experiments in Spanish and Mandarin Chinese. Spanish, an Indo-European language with rich verbal morphology and explicit lexical marking of intentional stance, may facilitate attitudinal distinctions through linguistic form. Mandarin Chinese, although fully capable of expressing intentionality, often relies more strongly on contextual and pragmatic inference during interpretation (Huang, 2015; Li and Thompson, 1981; Yao et al., 2021; Zhu and Wang, 2022). More broadly, research in linguistic relativity and bilingual cognition suggests that language structure may influence attentional processes, categorization, and social interpretation (Athanasopoulos et al., 2015; Boroditsky, 2011). Cross-cultural studies have also documented differences in interpersonal spacing norms, with Mediterranean cultures generally favoring greater physical proximity in social interaction than East Asian cultures (Beaulieu, 2004; Hall, 1966; Sorokowska et al., 2017). Because approach and avoidance orientations are grounded in embodied tendencies toward interpersonal proximity and withdrawal (Marrero et al., 2015, 2023a, 2023b), these cultural differences may influence how interpersonal closeness and distance are mentally simulated during narrative comprehension.

We addressed three primary questions. First, do approach-dominant episodes (Ap_Av_Ap) elicit greater perceived interpersonal closeness than avoidance-dominant episodes (Av_Ap_Av)? Second, does linguistic formulation modulate the processing and evaluation of interpersonal motivational episodes? Third, do these effects generalize across Spanish and Chinese speakers, or are they partially shaped by language- and culture-specific modes of interpersonal representation? Guided by this framework, we predicted that approach-dominant episodes would be evaluated as closer than avoidance-dominant ones through the cumulative integration of motivational information across the episode. We further predicted that motivational sequence would modulate reading time of the first and second sentences, whereas it has no effect in the final sentence where information about the situational model would be updated, in particular the updating of interpersonal closeness-distance in order to respond the subsequent question, a process plausibly similar to both types of episodic sequence. Finally, we predicted that attitudinal formulations, by affording more direct access to motivational meaning, would facilitate processing without altering the final relational evaluation.

## 2 Materials and methods

### 2.1 Participants

Study 1 (Spanish sample). Twenty native right-handed Spanish speakers (17 women, 3 men) participated. Participants were university students of University of La Laguna aged between 18 and 36 years (*M* = 20.1, SD = 4.4). The study was conducted in accordance with the Declaration of Helsinki and received ethical approval from the Institutional Ethics Committee of the University of La Laguna (CEIBA2023-3250). Right-handedness was assessed by the Edinburgh Handedness Inventory (Oldfield, 1971). An informed consent was obtained for each participant. Sample size was determined *a priori* using G*Power 3.1 (Faul et al., 2007; test family: t tests; test: difference between two dependent means, matched pairs; two-tailed; *α* = 0.05; power = 0.80). Assuming a medium-to-large within-participant effect (dz = 0.5–0.6), this analysis indicated a required sample of approximately 24–34 participants. The combined sample of 39 participants (20 Spanish, 19 Chinese) therefore exceeded the number required to detect the primary within-participant effects of Sequence and Formulation. Between-group (Language) effects and higher-order interactions, which require substantially larger samples, were correspondingly less well powered and are interpreted with caution.

Study 2 (Chinese sample). Twenty native Chinese speakers (16 women, 4 men) participated. All were university students aged between 17 and 31 years (*M* = 21.1, SD = 4.4) enrolled at Hengyang Normal University. Participation was voluntary and conducted under the same ethical standards and inclusion criteria as Study 1.

### 2.2 Design

A 2 × 2 × 2 factorial design was used with two within-subject factors: interaction sequence: Ap_Av_Ap (approach-avoidance-approach), Av_Ap_Av (avoidance-approach-avoidance), and linguistic formulation: attitudinal verb; e.g., “Juan asked you to join his group and you accepted him”) vs. behavioral expression; e.g., “Juan asked you to join his group and you said yes to him”). The between-subject factor was language context (Chinese vs. Spanish).

### 2.3 Materials

A total of 76 experimental narrative episodes were created in each language. The materials were evenly distributed across the four experimental conditions, resulting in 19 episodes per condition. Each episode consisted of three consecutive interactions between the protagonist (role assumed by the participant) and a fictional character. The interactions described everyday social behaviors either attitudinally or behaviorally formulated. For each episode, two parallel linguistic versions were constructed: Attitudinal version, explicitly encoding the protagonist’s internal stance (e.g., “you rejected him”), Behavioral version, describing the action without explicitly specifying the attitude (e.g., “you said no to him”). The two versions were matched in length, syntactic structure, and semantic content. To confirm that the attitudinal and behavioral versions described the same interactional events, the materials were reviewed by independent researchers at the University Institute of Neuroscience of University of La Laguna (Spain) and at Dalian University of Technology (China), who judged the two versions of each episode to be equivalent in the interaction they conveyed, differing only in whether the protagonist’s internal stance was made explicit. The Spanish and Chinese materials were constructed to preserve structural equivalence across languages. An example of one episode across the four experimental conditions in both languages is presented in Table 1. Lexical frequency was not controlled between the attitudinal and behavioral versions of the episodes. Although both versions were constructed to be comparable, differences in lexical frequency may have influenced reading times, and future studies should equate the two formulations using corpus-based frequency norms.

**Table 1:** Example of one narrative episode across experimental conditions in the Chinese and Spanish materials (English translations in parentheses).

Condition	Sentence 1	Sentence 2	Sentence 3
Chinese materials			
Ap_Av_Ap (attitudinal)	张晨在组会上提出了一个想法, 你支持了他。(Zhang Chen proposed an idea at the group meeting, and you supported him.)	你在食堂里遇到了张晨, 你忽视了他。(You met Zhang Chen in the cafeteria and ignored him.)	张晨在教室里请求加入学习小组, 你接受了他。(Zhang Chen asked to join the study group in the classroom, and you accepted him.)
Av_Ap_Av (attitudinal)	李航在组会上提出了一个想法, 你反对了他。(Li Hang proposed an idea at the group meeting, and you opposed him.)	你在食堂里遇到了李航, 你关心了他。(You met Li Hang in the cafeteria and showed concern for him.)	李航在教室里请求加入学习小组, 你拒绝了他。(Li Hang asked to join the study group in the classroom, and you rejected him.)
Ap_Av_Ap (behavioral)	王凯在组会上提出了一个想法, 你说很好。(Wang Kai proposed an idea at the group meeting, and you said “that’s good.”)	你在食堂里遇到了王凯, 你看向了其他方向。(You met Wang Kai in the cafeteria and looked in another direction.)	王凯在教室里请求加入学习小组, 你回答他可以。(Wang Kai asked to join the study group in the classroom, and you replied “yes.”)
Av_Ap_Av (behavioral)	刘胜在组会上提出了一个想法, 你说有问题。(Liu Sheng proposed an idea at the group meeting, and you said “there’s a problem.”)	你在食堂里遇到了刘胜, 你对他点了点头。(You met Liu Sheng in the cafeteria and nodded at him.)	刘胜在教室里请求加入你的学习小组, 你回答他不可以。(Liu Sheng asked to join your study group in the classroom, and you replied “no.”)
Spanish materials			
Ap_Av_Ap (attitudinal)	Carmen hizo una propuesta al grupo en la piscina y le apoyaste. (Carmen made a proposal to the group at the swimming pool, and you supported her.)	Cuando viste a Carmen la excluiste en la cafetería. (When you saw Carmen, you excluded her in the cafeteria.)	Carmen te ha pedido formar parte de tu grupo de amigos y la elegiste para tu grupo. (Carmen asked to join your group of friends, and you chose her for your group.)
Av_Ap_Av (attitudinal)	Aroa hizo una propuesta al grupo en la piscina y la ridiculizaste. (Aroa made a proposal at the swimming pool, and you ridiculed her.)	Cuando viste a Aroa la acogiste en la cafetería. (When you saw Aroa, you welcomed her in the cafeteria.)	Aroa te ha pedido formar parte de tu grupo de amigos y la descartaste para tu grupo. (Aroa asked to join your group of friends, and you rejected her.)
Ap_Av_Ap (behavioral)	Helena hizo una propuesta al grupo en la piscina y le aplaudiste. (Helena made a proposal at the swimming pool, and you applauded her.)	Cuando viste a Helena la apartaste en la cafetería. (When you saw Helena, you moved away from her in the cafeteria.)	Helena te ha pedido formar parte de tu grupo de amigos y le dijiste que sí para tu grupo. (Helena asked to join your group of friends, and you said yes.)
Av_Ap_Av (behavioral)	Candela hizo una propuesta al grupo en la piscina y te reíste de ella. (Candela made a proposal at the swimming pool, and you laughed at her.)	Cuando viste a Candela le hablaste en la cafetería. (When you saw Candela, you spoke to her in the cafeteria.)	Candela te ha pedido formar parte de tu grupo de amigos y le dijiste que no para tu grupo. (Candela asked to join your group of friends, and you said no.)

In addition to the three interaction sentences, each episode concluded with a question asking participants to evaluate their perceived interpersonal closeness toward the fictional character. Participants responded using a 5-point Likert scale: 1 = very distant, 2 = somewhat distant, 3 = neutral, 4 = somewhat close, 5 = very close.

### 2.4 Procedure

**Figure 1:**
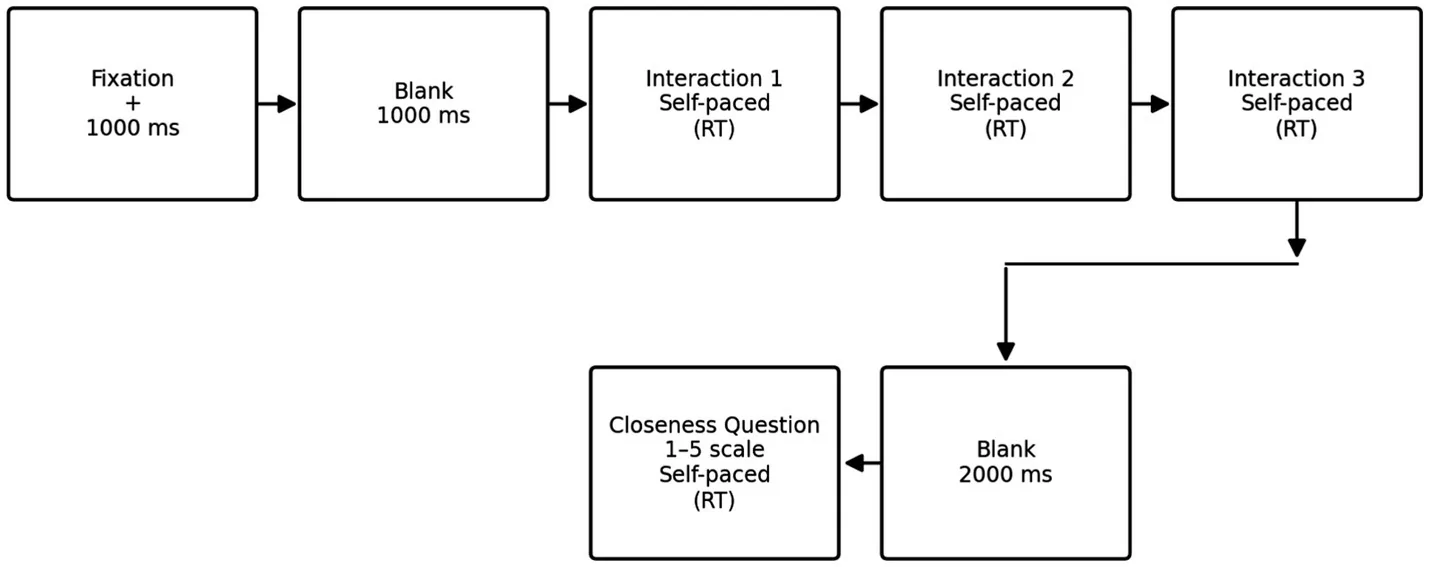
Schematic of the trial procedure. Each episode began with a central fixation cross (1,000 ms) followed by a blank screen. The three interaction sentences were then presented one at a time and self-paced, each remaining on screen until a key press, so that a reading time was recorded for each. After the third sentence, the interpersonal closeness-distance question appeared and remained on screen until the participant responded on a 1–5 scale.

The study was conducted individually in a controlled laboratory environment. Episodes were presented on a computer screen, and participants were seated approximately 60 cm from the screen. Participants were instructed to imagine themselves as the protagonist in each episode and to respond intuitively. They were told there were no right or wrong answers and that the goal was to assess their subjective perception of interpersonal closeness-distance. Each trial followed the same structure: Sentence 1 (first interaction), Sentence 2 (second interaction), Sentence 3 (third interaction), Interpersonal closeness question. The procedure for episodes display is shown in Figure 1. Stimuli were presented and reading times were recorded using Presentation software (Neurobehavioral Systems, Inc.). Each episode began with a central fixation cross displayed for 1,000 ms, followed by a brief blank screen. The three interaction sentences were then presented one at a time, each remaining on the screen until the participant pressed a key (self-paced), so that a reading time was recorded for each sentence. After the third sentence a blank screen was shown, and the closeness-distance question appeared 2,000 ms later, remaining on the screen until the participant responded on the 1–5 scale.

Reading times were recorded for each of the four segments (Sentence 1, Sentence 2, Sentence 3, Question). Episodes were presented in randomized order for each participant to avoid order effects. Three practice trials preceded the experimental trials and were excluded from analysis. The total duration of the session was approximately 30 min.

### 2.5 Data preprocessing

Practice trials were excluded. Only responses within the valid 1–5 scale were retained. Reaction times were processed as follows: Raw duration values were converted to milliseconds. RTs were log-transformed to reduce positive skew. Outliers were identified using a ± 2.5 standard deviation criterion computed for each participant across all conditions at each sentence position. This condition-blind approach avoids ambiguities associated with cell-level trimming (e.g., Tukey 1.5 × IQR applied within participant × sequence × formulation cells), and is widely adopted in psycholinguistic research. Outlier trials were removed prior to statistical analysis. The overall RT exclusion rate was: S1: 3.2%, S2: 3.0%, S3: 2.8%, Question: 3.3%.

### 2.6 Data analysis

Data were analyzed using linear mixed-effects models (LMMs) fitted with the *fitlme* function in MATLAB (Statistics and Machine Learning Toolbox), with *p*-values computed via Satterthwaite’s degrees-of-freedom approximation. For each dependent variable (Sentence 1, Sentence 2, Sentence 3, and Question reading times, and closeness-distance ratings), we fitted a model with sequence (approach-avoidance-approach vs. avoidance-approach-avoidance), formulation (attitudinal vs. behavioral), and language (Spanish vs. Chinese) as effects-coded fixed factors, along with all two-way and three-way interactions. For reading time variables, log-transformed latencies were used as the dependent variable to reduce positive skew. By-participant random intercepts and random slopes for sequence, formulation, and their interaction were included, along with by-item random intercepts; where a maximal model failed to converge, the random-effects structure was simplified following Bates et al. (2015) and Matuschek et al. (2017); in the present data, the maximal structure converged for all dependent variables. Model parameters were estimated using restricted maximum likelihood (REML). Estimated marginal means (EMMs) were computed for significant effects; pairwise contrasts were evaluated using 95% confidence intervals with Satterthwaite degrees of freedom.

One Chinese participant was excluded from the primary analysis due to implausibly fast reading times (*M* = 313 ms for S2, representing 11% of the group mean), indicating failure to engage with the materials.

## 3 Results

All analyses reported below are based on the 39 participants retained after exclusions (20 Spanish and 19 Chinese); a sensitivity analysis that additionally included the excluded participant yielded identical inferential conclusions. Table 2 shows Type III tests of fixed effects.

**Table 2:** Type III tests of fixed effects.

Effect	F	df₁	df₂	*p*
Closeness ratings				
Sequence	104.52	1	74.51	< 0.001***
Formulation	0.52	1	72.12	0.471
Language	36.23	1	36.98	< 0.001***
Seq × Form	1.46	1	73.83	0.230
Seq × Lang	2.29	1	37.12	0.139
Form × Lang	1.06	1	1794.94	0.304
Seq × Form × Lang	0.01	1	170.36	0.919
Sentence 1 reading time (log)				
Sequence	4.47	1	67.58	0.038*
Formulation	2.54	1	61.21	0.116
Language	16.26	1	36.99	< 0.001***
Seq × Form	1.61	1	63.94	0.209
Seq × Lang	1.88	1	153.77	0.173
Form × Lang	0.58	1	67.10	0.450
Seq × Form × Lang	3.47	1	80.93	0.066†
Sentence 2 reading time (log)				
Sequence	4.36	1	73.66	0.040*
Formulation	5.57	1	72.96	0.021*
Language	15.55	1	37.00	< 0.001***
Seq × Form	0.08	1	72.06	0.772
Seq × Lang	0.06	1	55.51	0.803
Form × Lang	2.32	1	47.86	0.134
Seq × Form × Lang	0.24	1	190.41	0.624
Sentence 3 reading time (log)				
Sequence	0.70	1	65.73	0.405
Formulation	12.34	1	69.87	< 0.001***
Language	9.52	1	36.99	0.004**
Seq × Form	0.27	1	64.64	0.604
Seq × Lang	2.03	1	39.26	0.162
Form × Lang	0.57	1	77.01	0.452
Seq × Form × Lang	1.76	1	55.78	0.190
Question reading time (log)				
Sequence	2.21	1	70.14	0.141
Formulation	1.67	1	47.89	0.203
Language	0.40	1	37.00	0.533
Seq × Form	0.003	1	44.12	0.959
Seq × Lang	8.73	1	838.97	0.003**
Form × Lang	1.63	1	64.11	0.207
Seq × Form × Lang	1.72	1	52.75	0.195

*N* = 39. RT models use log-transformed latencies. Models included by-participant random intercepts and random slopes for Sequence, Formulation, and their interaction, and by-item random intercepts; this maximal structure converged for all dependent variables (see Data analysis and Supplementary Table S2). *p < 0.05; **p < 0.01; ***p < 0.001; †*p* < 0.10.

### 3.1 Interpersonal closeness-distance ratings

The LMM on closeness-distance ratings revealed a significant main effect of sequence, *F* (1, 74.51) = 104.52, *p* < 0.001. As predicted, interpersonal distance in Ap_Av_Ap sequence was rated as closer (*M* = 3.06) whereas in Av_Ap_Av ones was rated as more distant (*M* = 2.20). There was also a significant main effect of language, *F* (1, 36.98) = 36.23, *p* < 0.001, with Chinese participants rating episodes as involving greater interpersonal closeness (*M* = 2.87) overall and Spanish participants rating them as more distant (*M* = 2.40). The main effect of formulation was not significant, *F* (1, 72.12) = 0.52, *p* = 0.471.

The sequence × language interaction was not significant, *F* (1, 37.12) = 2.29, *p* = 0.139, nor did any other interaction reach significance (all ps > 0.13). The magnitude of the sequence effect was thus comparable across the two language groups.

### 3.2 Sentence 1 reading times

For sentence 1 reading times (log-transformed), there was a significant main effect of sequence, *F* (1, 67.58) = 4.47, *p* = 0.038, and language, *F* (1, 36.99) = 16.26, *p* < 0.001. Spanish participants read more slowly overall (*M* = 5038 ms) than Chinese participants (*M* = 3694 ms). Reading time was longer in Av_Ap_Av (*M* = 4465 ms) than in Ap_Av_Ap (*M* = 4296 ms) sequence. That is, it took more time when the sequence started with avoidance interaction in contrast to approach. Notably, this effect emerged at a point where only the first behavior had been read, so at that moment the two conditions differed in the motivational orientation of the opening behavior rather than in the overall composition of the episode.

The three-way sequence × formulation × language interaction approached but did not reach significance, *F* (1, 80.93) = 3.47, *p* = 0.066, suggesting that the interplay between motivational sequence and formulation may differ across the two languages. Because this effect did not attain conventional significance, it is interpreted with caution and not developed further. No other main effects or interactions reached significance (ps > 0.05).

### 3.3 Sentence 2 reading times

For sentence 2 reading times (log-transformed), there were significant main effects of sequence, *F* (1, 73.66) = 4.36, *p* = 0.040, and formulation, *F* (1, 72.96) = 5.57, *p* = 0.021. Sentence 2 reading time was longer in Ap_Av_Ap (*M* = 3344 ms) than in Av_Ap_Av (*M* = 3140 ms) sequence. Likewise, behavioral formulation sentences elicited longer S2 reading times (*M* = 3339 ms) than attitudinal ones (*M* = 3144 ms). The main effect of language was also significant, *F* (1, 37.00) = 15.55, *p* < 0.001, with Spanish reading more slowly (*M* = 3702 ms) overall than Chinese participants (*M* = 2755 ms). No other interaction effects reached significance (*p*s > 0.05).

### 3.4 Sentence 3 reading times

For sentence 3 reading times (log-transformed), formulation had a significant main effect, *F* (1, 69.87) = 12.34, *p* < 0.001, with attitudinal eliciting shorter reading times (*M* = 3462 ms) than behavioral sentences (*M* = 3727 ms). Language was also significant, *F* (1, 36.99) = 9.52, *p* = 0.004. Like in sentences 1 and 2, Spanish participants spent more reading time (*M* = 3995 ms) than Chinese (*M* = 3168 ms). No other main effects nor interactions reached significance (*p*s > 0.05).

### 3.5 Question reading times

For question reading times (log-transformed), the sequence × language interaction was significant, *F* (1, 838.97) = 8.73, *p* = 0.003. Decomposition revealed that the sequence effect was significant only in the Spanish group (*Δ* = 0.13 log units, *p* = 0.002), where approach-avoidance-approach sequences elicited longer question reading times; in the Chinese group, the sequence effect was not significant (*p* = 0.402; see Table 3). Simple effects of language were not significant at either sequence level (both ps > 0.26). No other main effects nor interactions reached significance (ps > 0.05).

**Table 3:** EMMs and pairwise contrasts for significant interactions.

Question RT (log) — sequence × language					
Condition	Mean (log)	SE	df	95% CI (log)	Back-trans
Ap_Av_Ap × Spanish	6.833	0.120	37.6	[6.59, 7.08]	928 ms
Ap_Av_Ap × Chinese	6.851	0.123	37.6	[6.60, 7.10]	945 ms
Av_Ap_Av × Spanish	6.698	0.115	37.7	[6.46, 6.93]	811 ms
Av_Ap_Av × Chinese	6.888	0.118	37.6	[6.65, 7.13]	981 ms

Pairwise: sequence effect at each language.					
At Language	Diff (APP−AV)	SE	df	*p*	95% CI
Spanish	0.135	0.043	192.3	0.002 **	[0.049, 0.220]
Chinese	−0.037	0.044	205.2	0.402	[−0.125, 0.050]

EMMs and contrasts reported for significant interactions (*p* < 0.05) only. Back-trans = geometric mean in ms (exp of log EMM). **p < 0.01.

## 4 Discussion

The present study investigated how motivational sequencing in second-person verbally simulated interaction episodes shapes evaluations of interpersonal affective closeness-distance, and whether these effects are modulated by linguistic formulation and language context. Across parallel experiments in Spanish and Mandarin Chinese, the findings provide converging evidence that the motivational dynamics of interpersonal interactions strongly influence relational evaluation. Dominant-approach episodes (Ap_Av_Ap) elicited greater perceived closeness, whereas dominant-avoidance episodes (Av_Ap_Av) elicited greater perceived distance. These effects emerged consistently across linguistic formulations and language groups, suggesting that readers dynamically integrate motivational information during interpersonal simulation to construct affective representations of social relationships.

These findings are consistent with embodied simulation accounts of language comprehension, which propose that understanding actions, intentions, and emotions involves the reactivation of sensorimotor and affective representations associated with lived experience (Barsalou, 1999, 2008; Gallese, 2006, 2009; Morey et al., 2022; Zwaan, 2004). When readers process second-person interpersonal episodes, they are required to mentally adopt the protagonist’s perspective and simulate the consequences of approach and avoidance interactions. The present results support this interpretation by showing that interpersonal closeness-distance evaluations are systematically modulated by the motivational composition of the episode. Interpersonal closeness and distance may be represented using spatial schemas originally developed for physical navigation (Anderson, 2010; Yamakawa et al., 2009) in which approach is associated with movement toward relational proximity and avoidance with movement away from it (Marrero et al., 2015, 2023a, 2023b; Yamakawa et al., 2009).

Importantly, the findings further suggest that interpersonal evaluation emerges through a process of dynamic integration across temporally unfolding interactions rather than from isolated sentence-level processing. Critically, sequence had significant effect in reading time of the first and second sentence revealing greater reading time for avoidance-interaction in contrast to approach one. However, no effect of sequence was observed in third sentence reading time. The apparent reversal in the direction of the sequence effect between the first and second sentences is consistent with this account: at Sentence 1, the processing cost attaches to episodes that open with an avoidance behavior, which violates the default expectation of an affiliative opening; at Sentence 2, the cost attaches to the point at which the motivational orientation shifts relative to the representation established by the first sentence, producing an updating cost. Situation model and event segmentation theories propose that readers continuously update mental representations as events unfold, integrating incoming information into a coherent representation of the episode (Bailey et al., 2017; Gámez and Marrero, 2001; Kurby and Zacks, 2012; Zacks et al., 2007; Zwaan, 2025). This temporal profile, which is processing costs during the earlier, motivationally incongruent interactions but not at the final sentence, together with a robust effect on the end-of-episode judgment, is what event-segmentation accounts predict if readers update their relational event model as motivational information accrues and consolidate a global closeness-distance representation at the episode boundary, where the evaluation is required.

At the same time, the absence of sequence effects at the final sentence should be interpreted cautiously. Because participants explicitly evaluated interpersonal closeness-distance after each episode, motivational updating may have been strategically enhanced by task demands. Future studies using implicit paradigms or incidental processing tasks are needed to determine whether the integration of interpersonal closeness-distance occurs automatically during comprehension or is amplified by explicit evaluative goals.

Linguistic formulation did not modulate final interpersonal evaluations. Attitudinal and behavioral formulations yielded comparable closeness-distance judgments, indicating that relational evaluation primarily depended on the motivational structure of the episode rather than on surface linguistic form. Nevertheless, attitudinal formulations facilitated sentence processing, producing shorter reading times than behaviorally phrased interactions in the second and third sentences reading time. This dissociation suggests that linguistic formulation affects processing efficiency without fundamentally altering relational interpretation.

One possible explanation is that attitudinal verbs provide more direct access to motivational and intentional information. Verbs such as *accept, reject, or support* explicitly encode interpersonal stance and may therefore facilitate the construction of interpersonal situation models by reducing inferential demands (Gámez and Marrero, 2001; Marrero et al., 2015, 2017, 2023a, 2023b). By contrast, behavioral formulations such as *say yes* or *say no* may require readers to infer motivational meaning from contextual cues, increasing processing demands despite leading to similar relational outcomes. Thus, language may not determine the ultimate representation of interpersonal closeness-distance but may shape the efficiency with which motivational information is accessed and integrated.

More broadly, these findings reinforce the view that language does not merely describe social behavior but contributes to how interpersonal experience is cognitively organized. The comparable evaluative outcomes across formulations suggest that readers converge on similar relational interpretations despite differences in linguistic encoding, whereas the processing advantage for attitudinal formulations indicates that explicit intentional information may facilitate embodied interpersonal simulation.

Although the core motivational sequence effect generalized across languages, several cross-linguistic differences emerged. First, Chinese participants showed shorter reading times overall than Spanish participants across the episode. This difference likely reflects broader characteristics of reading systems rather than differences in interpersonal processing per se. The morphosyllabic nature of the Chinese writing system allows efficient visual recognition of lexical meaning and extensive parafoveal processing during reading (Perfetti et al., 2005; Yan et al., 2009). Although cross-language comparisons of reading speed are methodologically complex, skilled Chinese readers achieve highly efficient reading through rapid lexical access and optimized eye-movement patterns (Rayner et al., 2012). Accordingly, the observed latency differences should be interpreted cautiously and not necessarily as evidence of differences in motivational simulation.

More relevant to the present research, Chinese participants rated episodes as involving greater interpersonal closeness than Spanish participants overall. Under the random-slope specification, the magnitude of the sequence effect on ratings did not differ reliably between the two language groups, so this cross-linguistic difference concerns the overall level of the closeness ratings rather than a differential sensitivity to motivational sequence.

Likewise, Spanish participants showed longer response latencies when evaluating approach-dominant episodes (Ap_Av_Ap), suggesting greater cognitive engagement during judgments of interpersonal closeness. One possible interpretation is that Spanish participants may place greater weight on affiliative relational information, leading to more elaborated processing of interpersonal proximity. Notably, the direction of this rating difference runs counter to what cross-cultural proxemics would predict: although Mediterranean cultures tend to favor greater interpersonal physical proximity than East Asian cultures (Beaulieu, 2004; Hall, 1966; Sorokowska et al., 2017), here it was the Chinese participants who rated episodes as closer. A more parsimonious account is offered by cross-cultural differences in rating-scale response style. East Asian respondents tend to favor the midpoint of rating scales and to avoid extreme responses relative to Western respondents (Chen et al., 1995), and Chinese respondents in particular show fewer extreme rejecting responses, plausibly because extreme negative endorsements may threaten the relational harmony valued in collectivist contexts (Guo and Spina, 2019). Such moderacy could plausibly compress ratings toward the scale midpoint, inflating apparent closeness, a pattern consistent with the overall rating difference observed here. This account should be treated with caution, as response-style differences do not always alter cross-cultural mean comparisons (Chen et al., 1995); culturally grounded variation in how approach- and avoidance-oriented interactions are embodied and simulated may also contribute.

Taken together, the present findings are in agreement with an embodied and language-mediated account of interpersonal cognition in which approach and avoidance orientations are dynamically integrated across unfolding interactions to guide evaluations of interpersonal closeness and distance. Although the motivational sequence effect generalized across the two examined languages, cross-cultural variation suggests that embodied interpersonal simulation is flexible and may be shaped by culturally grounded interaction norms. More broadly, the results underscore the importance of considering motivational sequencing, linguistic formulation, and sociocultural context when investigating how humans mentally represent interpersonal relationships during language comprehension.

### 4.1 Limitations

Despite the theoretical and empirical relevance of the present findings, several limitations should be acknowledged. First, the sample size was relatively modest and composed exclusively of university students, which may limit the generalizability of the results to broader age groups and sociocultural populations. Although linear mixed-effects modeling increases statistical robustness by accounting for item- and participant-level variability, replication with larger and more heterogeneous samples is necessary to strengthen external validity. A further limitation concerns the design itself: because the two sequences (Ap_Av_Ap and Av_Ap_Av) differ not only in the ordinal position of approach and avoidance behaviors but also in their overall motivational composition, the present contrast cannot fully isolate sequencing from composition. Future studies could hold the number of approach and avoidance behaviors constant while varying only their position (e.g., Ap_Ap_Av vs. Ap_Av_Ap vs. Av_Ap_Ap), or use sequences with an even number of behaviors to balance approach and avoidance frequency, to provide a cleaner test of order effects.

Second, lexical frequency and familiarity were not controlled between attitudinal and behavioral formulations. Although both versions of the episodes were matched in semantic content, syntactic structure, and length, differences in lexical accessibility may have partially contributed to the processing facilitation observed for attitudinal formulations. Future research should include normative lexical controls or corpus-based matching procedures to disentangle conceptual effects of motivational encoding from lexical processing differences.

Third, the present study relied exclusively on behavioral measures (reading times and evaluative ratings), which limits conclusions about the neurocognitive mechanisms underlying embodied simulation and interpersonal distance representation. Future work combining behavioral paradigms with neurophysiological or neuroimaging methods, such as electroencephalography (EEG), eye tracking, or non-invasive brain stimulation, may clarify the temporal and neural dynamics of motivational updating during second-person interaction episodes.

Fourth, the task explicitly required participants to evaluate interpersonal closeness-distance after each episode, making this dimension highly task-relevant. Consequently, it remains unclear whether the updating of affective closeness-distance occurs automatically during language comprehension or whether it is strategically enhanced by explicit evaluative goals. Experimental paradigms involving implicit measures or incidental processing tasks may help determine whether motivational integration emerges spontaneously during situation-model construction.

Finally, although the comparison between Spanish and Mandarin Chinese offers an important opportunity to examine linguistic and cultural modulation, the present design cannot fully disentangle language-related from culture-related effects. Differences observed between groups may reflect language structure, sociocultural norms of interpersonal distance, or both. In addition, participants’ second-language experience and degree of cross-cultural contact were not assessed, and these factors may have shaped how Chinese participants simulated the Spanish cultural context. Cross-cultural bilingual designs or within-subject multilingual paradigms may help clarify the relative contribution of linguistic encoding and cultural experience to motivational simulation in interpersonal narratives.

### 4.2 Contributions

The present study makes several theoretical and methodological contributions to research on social cognition, embodied language processing, and interpersonal simulation. First, it extends embodied simulation approaches to the domain of second-person verbally simulated interaction episodes by demonstrating that motivational sequencing of approach and avoidance behaviors systematically modulates perceived interpersonal closeness-distance. These findings suggest that readers dynamically integrate motivational information across temporally unfolding interactions to construct affective representations of interpersonal relationships. In particular, approach and avoidance dynamics of interaction may be represented using spatial schemas originally developed for spatial navigation: approach is associated with movement toward interpersonal closeness, whereas avoidance is associated with movement toward interpersonal distance.

Second, the study contributes to situation-model and event-segmentation theories by showing that motivational information is processed dynamically during episode unfolding while final relational evaluation appears to rely on an updating process occurring at the end of the episode. The absence of sequence effects in final sentence reading time, combined with robust effects on evaluative judgments, supports the idea that interpersonal closeness-distance is computed through integration of sequential motivational information rather than through local sentence-level processing alone.

Third, the present findings advance understanding of how linguistic formulation shapes interpersonal cognition. Although attitudinal and behavioral formulations produced comparable closeness-distance evaluations, attitudinal formulations facilitated processing speed, suggesting that verbs explicitly encoding intentional stance may support more efficient construction of interpersonal situation models. This finding reinforces the idea that language does not merely describe social behavior but may facilitate access to motivational representations embedded in interpersonal cognition.

Finally, the inclusion of parallel Spanish and Mandarin Chinese experiments provides novel cross-linguistic evidence regarding the robustness and flexibility of embodied interpersonal simulation. The motivational sequence effect generalized across languages, supporting the existence of shared cognitive mechanisms, while cultural-linguistic differences in processing speed and interpersonal evaluation suggest that embodied social simulation may also be modulated by culturally shaped interaction norms and linguistic encoding practices.

### 4.3 Conclusion

The present study demonstrates that approach and avoidance motivational orientations dynamically shape the evaluation of interpersonal affective closeness-distance in second-person verbally simulated interaction episodes. Across Spanish and Chinese participants, sequences containing more approach behaviors promoted evaluations of greater interpersonal closeness, whereas sequences dominated by avoidance promoted greater perceived distance. These findings support the view that readers construct relational representations through embodied simulation of approach motivational dynamics unfolding across time: approach would be represented as movement toward interpersonal closeness and avoidance toward distance. Practically, the sensitivity of relational evaluation to motivational sequencing may carry implications for the sequential design of interpersonal discourse in second-language teaching materials.

Importantly, linguistic formulation modulated processing efficiency but not final interpersonal evaluation. Attitudinal formulations facilitated sentence processing, suggesting more direct access to motivational and intentional information, whereas both attitudinal and behavioral formulations converged on similar relational judgments. This pattern indicates that interpersonal closeness-distance evaluation depends primarily on the motivational structure of the episode rather than on surface linguistic form.

At the same time, cross-linguistic differences suggest that motivational simulation is not entirely invariant across sociocultural contexts. Although the core sequence effect generalized across languages, Chinese participants rated episodes as involving greater interpersonal closeness overall, and Spanish participants showed greater latency during evaluation of approach sequences, suggesting differences in how interpersonal closeness and distance are cognitively weighted.

Taken together, these findings support an embodied and language-mediated account of interpersonal cognition in which approach and avoidance orientations are integrated into dynamic situation models that guide affective evaluation of social relationships. More broadly, the results underscore the importance of considering motivational sequencing, linguistic formulation, and sociocultural context when investigating how humans mentally simulate interpersonal interactions.

## Data Availability

The raw data supporting the conclusions of this article will be made available by the authors, without undue reservation.
