# Supplementary material for: Approach-avoidance sequencing shapes embodied simulation of interpersonal closeness-distance: evidence from second-person episodes in Spanish and Chinese
Source: Front Psychol. 2026 Aug 21;17:1925565. doi: 10.3389/fpsyg.2026.1925565 (PMC13541451; doi:10.3389/fpsyg.2026.1925565)
Supplement: Supplementary file 1 [file Table_1.DOCX]

**Supplementary Material**

**Supplementary Tables**

*Table S1. Mean reading times (ms) and closeness–distance ratings by condition (N = 39).*

| **Language** | **Sequence** | **Formulation** | **Sentence 1** | **Sentence 2** | **Sentence 3** | **Question** | **Rating** |
| --- | --- | --- | --- | --- | --- | --- | --- |
| Spanish | Ap_Av_Ap | attitudinal | 4982 | 3685 | 3789 | 1413 | 2.80 |
| Spanish | Ap_Av_Ap | behavioral | 5053 | 3939 | 4206 | 1430 | 2.95 |
| Spanish | Av_Ap_Av | attitudinal | 4993 | 3412 | 3940 | 1238 | 1.92 |
| Spanish | Av_Ap_Av | behavioral | 5126 | 3781 | 4042 | 1241 | 1.93 |
| Chinese | Ap_Av_Ap | attitudinal | 3362 | 2856 | 3007 | 1434 | 3.20 |
| Chinese | Ap_Av_Ap | behavioral | 3738 | 2844 | 3228 | 1546 | 3.29 |
| Chinese | Av_Ap_Av | attitudinal | 3896 | 2583 | 3072 | 1525 | 2.52 |
| Chinese | Av_Ap_Av | behavioral | 3782 | 2742 | 3370 | 1628 | 2.45 |

*Note. Cell means are arithmetic means computed on the analyzed (±2.5 SD trimmed) trials. Reading times are in milliseconds; ratings are on a 1–5 closeness–distance scale. Inferential tests were conducted on log-transformed reading times using the linear mixed-effects models reported in the main text.*

*Table S2. Sensitivity of fixed-effect significance (p-values) to the random-effects structure (primary sample, N = 39).*

| **Effect** | **M0** | **M1** | **M2** | **M3** | **M4 (primary)** |
| --- | --- | --- | --- | --- | --- |
| ***Sentence 1 (log RT)*** | | | | | |
| Sequence | .036 | .036 | .036 | .036 | .038 |
| Formulation | .104 | .104 | .104 | .106 | .116 |
| Language | <.001 | <.001 | <.001 | <.001 | <.001 |
| Seq × Form | .194 | .194 | .194 | .194 | .209 |
| Seq × Lang | .158 | .160 | .158 | .160 | .173 |
| Form × Lang | .419 | .419 | .419 | .424 | .450 |
| Seq × Form × Lang | .047 | .047 | .047 | .047 | .066 |
| ***Sentence 2 (log RT)*** | | | | | |
| Sequence | .035 | .037 | .037 | .039 | .040 |
| Formulation | .018 | .018 | .021 | .021 | .021 |
| Language | <.001 | <.001 | <.001 | <.001 | <.001 |
| Seq × Form | .767 | .770 | .766 | .769 | .772 |
| Seq × Lang | .759 | .774 | .774 | .796 | .803 |
| Form × Lang | .082 | .083 | .125 | .139 | .134 |
| Seq × Form × Lang | .627 | .624 | .623 | .618 | .624 |
| ***Sentence 3 (log RT)*** | | | | | |
| Sequence | .380 | .404 | .404 | .404 | .405 |
| Formulation | .001 | .001 | .001 | .001 | .001 |
| Language | .004 | .004 | .004 | .004 | .004 |
| Seq × Form | .605 | .607 | .606 | .601 | .604 |
| Seq × Lang | .098 | .158 | .158 | .160 | .162 |
| Form × Lang | .418 | .421 | .432 | .449 | .452 |
| Seq × Form × Lang | .169 | .168 | .168 | .171 | .190 |
| ***Question (log RT)*** | | | | | |
| Sequence | .140 | .142 | .140 | .147 | .141 |
| Formulation | .167 | .169 | .167 | .182 | .203 |
| Language | .534 | .534 | .534 | .534 | .533 |
| Seq × Form | .957 | .953 | .957 | .954 | .959 |
| Seq × Lang | .003 | .003 | .003 | .004 | .003 |
| Form × Lang | .164 | .165 | .164 | .182 | .207 |
| Seq × Form × Lang | .143 | .142 | .143 | .143 | .195 |
| ***Rating*** | | | | | |
| Sequence | <.001 | <.001 | <.001 | <.001 | <.001 |
| Formulation | .475 | .471 | .471 | .472 | .471 |
| Language | <.001 | <.001 | <.001 | <.001 | <.001 |
| Seq × Form | .230 | .227 | .227 | .227 | .230 |
| Seq × Lang | .004 | .137 | .137 | .137 | .139 |
| Form × Lang | .328 | .306 | .306 | .305 | .304 |
| Seq × Form × Lang | .927 | .928 | .928 | .927 | .919 |

*Note. p-values are from Type III tests with Satterthwaite degrees of freedom, estimated by REML; all models included by-participant and by-item random intercepts. M0 = random intercepts only (the structure of the originally submitted analysis; this column reproduces the originally reported results). M1 = M0 plus a by-participant random slope for Sequence; M2 = M0 plus uncorrelated (zero-covariance) by-participant random slopes for Sequence and Formulation; M3 = M0 plus correlated by-participant random slopes for Sequence and Formulation; M4 = the primary specification reported in the main text (M3 plus a by-participant random slope for the Sequence × Formulation interaction; see Table 2). All models converged; for Sentence 1, Question, and Rating, the M2 fit lay at the boundary of the parameter space (a random-slope variance estimated near zero) and should be interpreted with corresponding caution. Every main effect, and the Sequence × Language interaction on Question reading times, retains the same significance status under every structure. Two higher-order interactions are structure-dependent: the Sequence × Language interaction on Rating is significant only without random slopes (M0: p = .004; p ≥ .137 under all slope structures), and the Sequence × Formulation × Language interaction on Sentence 1 lies at the significance threshold across structures (p = .047–.066), reaching non-significance under the primary specification; both are therefore treated as unreliable and are not interpreted in the main text.*
